# ECG Approximate Entropy in the Elderly during Cycling Exercise †

**DOI:** 10.3390/s22145255

**Published:** 2022-07-14

**Authors:** Jiun-Wei Liou, Po-Shan Wang, Yu-Te Wu, Sheng-Kai Lee, Shen-Da Chang, Michelle Liou

**Affiliations:** 1 Department of Electrical Engineering, Ming Chi University of Technology, New Taipei City 243, Taiwan; jwliou49908@mail.mcut.edu.tw; 2 Department of Neurology, Municipal Gandau Hospital, Taipei 112, Taiwan; b8001071@yahoo.com.tw; 3 Institute of Biophotonics, National Yang-Ming Chiao Tung University, Taipei 112, Taiwan; ytwu@nycu.edu.tw; 4 Taiwan International Graduate Program in Interdisciplinary Neuroscience, National Cheng-Kung University & Academia Sinica, Taipei 701, Taiwan; dermrian@stat.sinica.edu.tw; 5 Institute of Statistical Science, Academia Sinica, Taipei 115, Taiwan; mike0711@me.com

**Keywords:** aging, cycling exercise, EEG oscillations, ECG approximate entropy

## Abstract

Approximate entropy (ApEn) is used as a nonlinear measure of heart-rate variability (HRV) in the analysis of ECG time-series recordings. Previous studies have reported that HRV can differentiate between frail and pre-frail people. In this study, EEGs and ECGs were recorded from 38 elderly adults while performing a three-stage cycling routine. Before and after cycling stages, 5-min resting-state EEGs (rs-EEGs) and ECGs were also recorded under the eyes-open condition. Applying the K-mean classifier to pre-exercise rs-ECG ApEn values and body weights revealed nine females with EEG power which was far higher than that of the other subjects in all cycling stages. The breathing of those females was more rapid than that of other subjects and their average heart rate was faster. Those females also presented higher degrees of asymmetry in the alpha and theta bands (stronger power levels in the right frontal electrode), indicating stressful responses during the experiment. It appears that EEG delta activity could be used in conjunction with a very low ECG frequency power as a predictor of bursts in the heart rate to facilitate the monitoring of elderly adults at risk of heart failure. A resting ECG ApEn index in conjunction with the subject’s weight or BMI is recommended for screening high-risk candidates prior to exercise interventions.

## 1. Introduction

Regular exercise can enhance cardiac function and counteract muscle weakness and physical frailty in the elderly, diminishing the effect of afflictions such as muscle atrophy [1,2,3]. Exercise has also been shown to have positive effects on motor functions, auditory attention, cognitive speed, and visual attention [4,5]. Heart rate variability (HRV) indices derived from ECGs have been recommended as a predictor of physiological distress during exercise [6,7]. One study reported that physical exercise can have beneficial effects for cardiac autonomic nervous function and offset the negative impact due to obesity on HRV [8]. The connection between HRV and exercise needs to be carefully examined because of the effects of gender and age as confounders in HRV indices. Subjects 40–80 years old present a marked decrease in inter-beat intervals (RR intervals) in the HF% (0.15–0.40 Hz) and LF% (0.04–0.15 Hz) ranges. However, LF% is significantly higher than HF% in subjects 40–60 years old, particularly in men [9]. Serum testosterone levels are positively correlated with several HRV indices in the time domain, such as SDNN (standard deviation of RR intervals) and RMSSD (root mean square of successive differences in RR intervals) [10]. Men above the age of 60 often present an imbalance between testosterone and estradiol levels, affecting autonomic functions with higher LF% values and higher LF/HF ratios which are strongly associated with the risk of heart failure [11]. Many patients with a chronic heart disability develop periodic breathing patterns (i.e., clusters of breaths separated by intervals of no breathing), which generates very low frequency (VLF; 0–0.04 Hz) rhythms in the heart rate [12,13].

Nonlinear indices are also important measures of autonomic functions. Approximate entropy (ApEn) and sample entropy (SampEn) are two nonlinear indices commonly used to measure the nonlinear aspects of RR intervals [14]. Although the precise meaning of the two entropy indices has yet to be elucidated, it is believed that a reduction in entropy is related to the autonomic pathology and decreased RR interval complexity, particularly in aged people [15,16,17]. Entropy indices are seen as a potential marker by which to assess the benefits of exercise [7,14,18,19]. In one study, the SDNN, RMSSD, ApEn and SampEn values of patients with chronic heart failure were shown to be significantly lower than those of healthy controls during walking [20]. In another study, the ApEn values of 250 patients with heart failure (≥65 years old) were significantly lower than that of 100 age-matched controls in ECGs recorded over a period of 24 h [21]. Nevertheless, researchers have reported a general increase in ApEn and SampEn indices as a function of age (from 30 to 79 years old) in patients with congestive heart failure [22].

EEG activity has been used to reflect affective, perceptual and cognitive changes of exercise [23]. There is a notable increase in EEG power with the onset of fatigue during high-intensity cycling exercises, with the most pronounced increase in the frontal cortex [24,25]. In instances of severe fatigue, it appears that the average power within each frequency band increases with the duration of exercise, whereas the power ratio (theta + alpha)/beta gradually decreases from exercise initiation until the subject rests after the exercise is concluded [26]. Gamma activity has been shown to play a role in motor control (e.g., encoding information related to limb movement) [27]. The gamma and theta ratio (GTR) has been proposed as a marker for stress [28]. However, meta-analysis on studies linking changes in EEG patterns and exercise interventions has revealed that alpha and beta activity tended to be overrepresented in those studies. It is also true that variations in the definition of frequency bands made it difficult to replicate the findings in those studies [29]. It has been reported that EEG power spectral density is higher under exercise conditions than under resting-state conditions and ECG HF% is significantly lower [30]. One study reported increases in theta, alpha and beta power levels in the frontal region after an open-loop graded exercise test on a bicycle ergometer. The same study also reported a significant decrease in HRV frequency parameters after the test [31]. Subjects presenting EEG asymmetry (left and right sides) tend to exhibit greater emotional responsiveness than those without such asymmetry. One study on athletes with a history of concussion reported positive correlations between alpha asymmetry (i.e., relatively greater right frontal activation) and self-reported depression and anxiety, as well as positive correlations between beta asymmetry and self-reported anger/aggression under resting-states [32]. Among the elderly, beta asymmetry is positively associated with scores on the Total Mood Disturbance questionnaire [33].

Aging is associated with a variety of geriatric issues, including frailty [34], which involves the decline of several physiological systems with gradually worsening health outcomes and an increased risk of hospitalization [35]. Among the elderly, regular exercise has benefits for cardiac function, cognitive ability and overall health. However, it is important to monitor the risk of heart failure during exercise. Numerous researchers have examined the link between ECG and EEG patterns under the exercise and resting-state conditions. In the current study, we explored the possibility of using resting-state HRV as a physiological marker for EEG oscillatory responses and HRV in elderly adults during exercise. Our study focused on the approximate entropy index under pre-exercise resting-state conditions, due to its potential role in revealing breathing patterns and cardiovascular conditions, particularly in the elderly [36]. In this exploratory study, we classified thirty-eight elderly adults into three groups according to pre-exercise resting ECG ApEn and weight values, as a basis by which to compare EEG and HRV features during cycling. We also discuss the implications of these features in terms of heart failure during exhaustive exercise.

## 2. Materials and Methods

### 2.1. Subjects and Experimental Protocol

Thirty-eight elderly adults were recruited for the experiment, including 25 females (averaged age 66.04 ± 5.1 yrs, height 153.3 ± 5.9 cm, weight 57.96 ± 8.2 kg) and 13 males (averaged age 65.85 ± 6.2 yrs, height 167.89 ± 3.9 cm, weight 73.89 ± 11.5 kg). Two males had BMI scores within the range of obesity (BMI scores slightly > 30). Other subjects were within the normal range (18.5 < BMI < 24.9) or overweight range (25 < BMI < 29.9). None of the subjects presented cardiovascular or chronic diseases, and none had previous experience with cycling as a form of exercise. All subjects provided informed consent, and the study was approved by the Institutional Review Board (IRB) at Municipal Gandau Hospital. Prior to the experiment, the subjects obtained approval from a professional nurse in the Health Center of the hospital ensuring that their psychological and physiological conditions were sufficient to endure the cycling exercise without incident. The experiment was performed between 9 and 12 a.m., and subjects were requested to have breakfast before visiting the Health Center in which the experiment was performed. The experiment involved two exercise sessions: the warm-up session followed by the experiment session. The warm-up session involved a 40-s exercise period of pedaling a spin bike (Well-Come XR-G5, Taoyuan, Taiwan) without resistance, followed by a 20-s resting period of no pedaling. This cycling section was repeated 10 times, during which the cycling load was fixed at level 3 out of 10 (maximum), as determined in a previous experiment [37]. The experiment session began with the capture of 5-min resting-state EEG (rs-EEG) and ECG (rs-ECG) recordings under the eyes-open condition, followed by the same recording procedure during 3 cycling stages, each of which comprised a 5-min cycling period and a 30-s resting period. As in the warm-up session, the cycling load was fixed at level 3 without resistance. The experiment session ended with the capture of 5-min rs-EEG and rs-ECG recordings. Note that the EEGs and ECGs obtained during the experiment session will be hereafter referred to as Rest-1, Cycling-1, Cycling-2, Cycling-3 and Rest-2.

### 2.2. EEG and ECG Recording

Data acquisition was performed as per the guidelines of the IRB Human Ethics Committee at Municipal Gandau Hospital. All EEGs and ECGs, along with EOGs and EMGs, were recorded using Brain Products Active Wet electrodes (Brain Products, Gilching, Germany) with impedance guided by data quality, as suggested in the manual provided by Brain Products. EOGs and EMGs were used to minimize artifacts in EEG signals due to muscle activity and eyes blinking. All electrodes were arranged in accordance with the 10–20 system (C2, C4, Cz, F3, F4, Fz, P3, P4, Pz). A ground electrode was used, embedded in the cap at the Fpz position. EEGs were recorded online and rereferenced to the electrode A1 clipped to the left earlobe. The results were digitized at 1000 Hz at 24-bit resolution. Signals were filtered using an online bandpass filter with a cutoff of 0.1–100 Hz. In addition to the 9 EEG electrodes, 1 ground and 1 reference electrode, we recorded vertical and horizontal bipolar EOGs from passive Ag/AgCl easy cap disk electrodes affixed above and below the left eye. EEG and EOG signals were amplified using a V-AMP 16-channel amplifier (Brain Products). Bipolar ECGs were recorded from the anterior (right clavicle) and lateral (between left 8th and 9th ribs) chest regions using A2 clipped to the right earlobe as the ground electrode. Surface EMG electrodes were placed on the quadriceps muscle separated by 10-cm with an additional electrode placed on the kneecap for recording ground signals. The impedance of all electrodes was controlled to be below 20 kΩ during the experiment.

### 2.3. Signal Processing

On-going EEG time series were pseudo-epoched using a 5-s interval. Artifacts resulting from eye movements, blinks, muscle activity and line noises were estimated using independent component analysis (ICA) in EEGLab [38]. We differentiated brain activity from artifacts using an automated method based on the reference time series in EOGs, ECGs and EMGs, which served as regressors in multiple regression analysis. Given an $R^{2}$ value > 0.5, the corresponding ICA component scores were corrected by removing artifacts predicted by the time series in regressors. Clean EEGs were then reconstructed by multiplying the corrected component-score matrix with the estimated weighting matrix derived from ICA. The clean EEGs were applied to time–frequency analysis and the Morlet wavelet transform was applied to time–frequency representation of on-going EEGs. After preprocessing, EEG power spectral analysis was, respectively, performed on the 0.5–50 Hz interval within each channel, subject and experimental stage. The window size used was 512 samples (512-ms) for the lowest frequency. A total of 500 frequencies between 0.5 and 50 Hz were displayed and oscillatory activity was extracted in the delta (0.5−3 Hz), theta (3.5−7 Hz), alpha (8−13 Hz), beta (15−24 Hz) and gamma (28−48 Hz) bands [39,40,41,42]. ECGs were processed to derive HRV indices using Kubios HRV-2.2 software [43]. Time–domain indices included those calculated on the basis of RR-intervals. Frequency–domain HRV indices were computed using power spectral analysis, wherein the smoothed RR-interval time series was transformed into the frequency–domain. Non-linear indices were also computed using the RR-interval time series.

### 2.4. Approximate Entropy

Entropy indices measure the amount of information required to predict the future state of a system, that is, to summarize unpredictability of a system. The ApEn value is inversely proportional to the likelihood that runs of patterns that are close remain close in the following incremental comparisons [44]. Given an RR interval time series $u_{1},\;u_{2}$, …, $u_{N}$, we define a run of length m to be $X_{m}\left(i\right)$ = [$u_{\left(i\right)},\;u_{\left(i+1\right)}$, …, $u_{\left(i+m-1\right)}]$ for 1 ≤ i ≤ *N* − *m* + 1. The distance between $X_{m}\left(i\right)$ and $X_{m}\left(j\right)$ is defined as d$\left[X_{m}\left(i\right),\;X_{m}\left(j\right)\right]=\mathrm{Max}\left\{\left|u\left(i+k\right)-u\left(j+k\right)\right|:0\leq k\leq m-1\right\}$, which is the maximum absolute difference between their corresponding scalar elements. In addition, let

$$C_{i}^{m}(r)=\left\{\mathrm{Number}\;\mathrm{of}\;X_{m}\left(j\right)\;\mathrm{with}\;d\left[X_{m}\left(i\right)\;,\;X_{m}\left(j\right)\right]\;\leq r\right\}∕\left(N-m+1\right),$$

and

$$\Phi^{m}\left(r\right)=\sum_{i}^{N-m+1}ln\left(C_{i}^{m}\left(r\right)\right)∕\left(N-m+1\right).$$

The ApEn index with parameters (*m*, *r*, *N*) is equal to the difference between $\Phi^{m}\left(r\right){\;\mathrm{and}\;\Phi }^{m+1}\left(r\right)$. The ApEn algorithm counts self-matching to avoid the occurrence of *ln*(0) in the calculations. The index has two well-known shortcomings. First, it depends on *N* and is generally lower than expected when dealing with short records. Second, it lacks consistency. For example, if the ApEn of one dataset is higher than that of another, then it is unnecessarily higher for all conditions tested. The SampEn index is a large sample approximation of ApEn [45,46], which means that SampEn is the negative natural logarithm of the ratio between $A^{m+1}\left(r\right)$ and $B^{m}\left(r\right)$, where $A^{m+1}\left(r\right)$ indicates the proportion of distances between runs of size *m* + 1 which are smaller than or equals to *r* and $B^{m}\left(r\right)$ is defined analogously. The range of ApEn and SampEn indices are between 0 and positive values, where a smaller value indicates more self-similarity in the time series. As a measure of HRV, *m* is commonly taken to be 2 and *r* is selected as 2*SDNN. It has been found that SampEn is more reliable than ApEn for short-term RR-interval time series and less sensitive to changes in N [47]. The SampEn index is generally recommended for experiments on clinical patients with a variety of RR-interval lengths [22]. When ECGs are recorded during exercise, ApEn tends to be more stable than SampEn in reflecting complexity in the heart rate. Nonetheless, the two indices provide similar classification results when dealing with elderly adults under resting-state conditions, as described in the next section. 

### 2.5. Statistical Classification Analysis

EEG power spectral density was summarized by averaging power levels within individual resting and cycling stages, respectively. This study was exploratory in nature with no clinical patients for comparison. Aged but healthy subjects were grouped according to their Rest-1 ApEn values. The Rest-1 ApEn values and EEG in different frequency bands were initially treated as covariates in repeated measure ANOVA to account for differences among subjects in their EEG power levels during cycling and Rest-2 stages. In ANOVA analysis, EEG power levels and ApEn values within Rest-1 were significantly associated with EEG power levels during cycling stages and Rest-2. For instance, changes in the frontal delta power levels (average of power levels in F3, Fz and F4) across cycling and Rest-2 stages were significantly associated with Rest-1 ApEn values (*p* = 0.012) and Rest-1 frontal delta (*p* < 0.001). The interaction between ApEn and the subject’s weight was also significantly associated with EEG power levels for all frequency bands during cycling (e.g., *p* = 0.015 for frontal delta). Note that the height of subjects was not significantly interacted with ApEn. In fact, ApEn (or SampEn) in Rest-1 was the only HRV measure, among time–domain, frequency–domain, and nonlinear HRV indices, that showed a predictive power for EEG activity in cycling stages and Rest-2, considering that Rest-1 EEG power levels were already in the ANOVA model. Our study used the K-mean classifier to group subjects according to their ApEn values in Rest-1 and body weights; both variables were transformed into Tukey’s normalized scores before classification. In the sequel, the results and discussion sections mainly focus on EEG and HRV features in the three classes, and the implication of these features for predicting the risk of heart failure in subjects during cycling exercise.

## 3. Results

**Figure 1:**
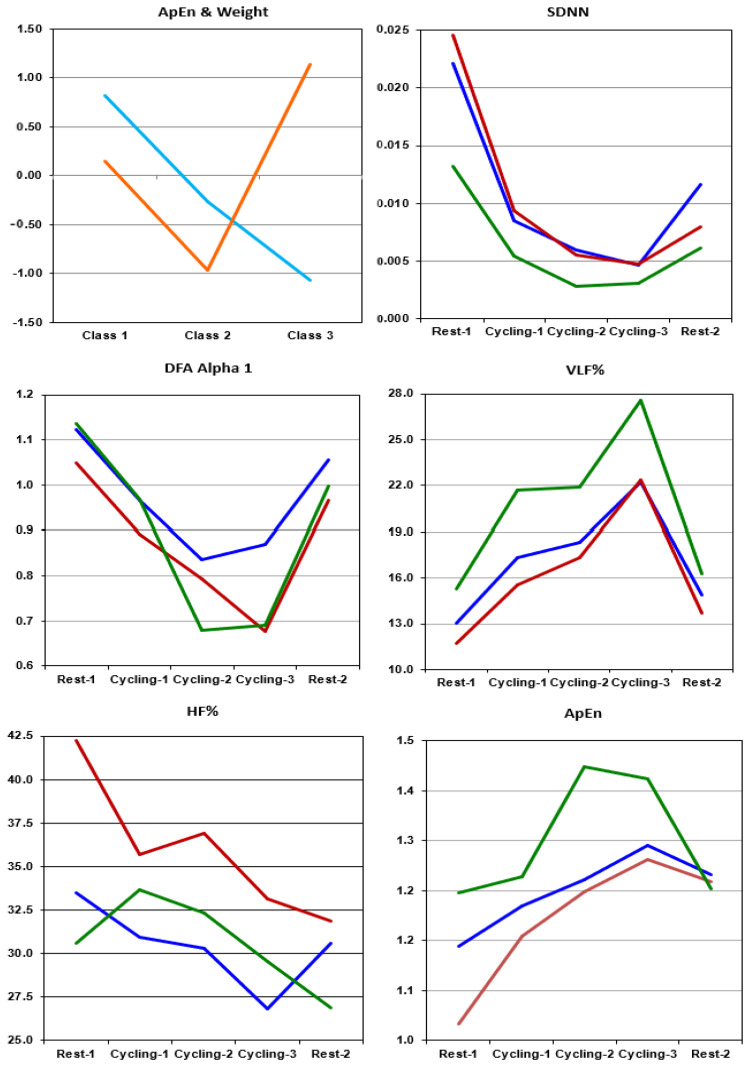
Normalized ApEn values and weight scores in the 3 classes as well as time–domain, frequency–domain and nonlinear HRV indices during Rest-1, Cycling-1, Cycling-2, Cycling-3 and Rest-2. At the top of left panel: **cyan** denotes normalized scores of weights and **orange** denotes normalized scores of ApEn values, respectively. The plots for HRV indices are shown in **blue** for Class 1, **red** for Class 2 and **green** for Class 3.

The oscillatory activity of females was stronger than that of males in all frequency bands throughout the five experimental stages. The increases in the ApEn values for females were more pronounced than those of males during the cycling stages. Three classes showed significant differences in normalized body-weight (*p* < 0.001) and ApEn scores (*p* < 0.001). Figure 1 plots the average normalized scores of the two variables in the three classes. Class 1 comprised eight females and eight males (average age: 65.50 ± 4.23 yrs; average weight 73.34 ± 10.22 kg). Class 2 comprised eight females and five males (average age: 65.46 ± 6.53 yrs; average weight: 59.54 ± 6.61 kg). Class 3 comprised nine females (average age: 67.56 ± 5.94 yrs; average weight: 51.33 ± 4.74 kg) who had an average weight lower than that of the other subjects. Figure 1 also plots a few HRV indices for the three classes across all experimental stages. The SDNN index presented a decrease between Cycling-1 and Cycling-3 and a slightly increase during Rest-2. Other time–domain indices presented patterns similar to those of the SDNN index, which indicates that heart rates were faster during cycling stages than during resting conditions (or that the subjects tended to be under sympathetic control).

Figure 1 plots the detrended fluctuation analysis (DFA) of the alpha1 indices which determined the short term (alpha1: 4–12 beats) correlations between successive heartbeats. A DFA-alpha1 of 1.0 at rest is an indication of normalcy, whereas any deviation from 1.0 indicates elevated randomness during moderate-to-high intensity exercise. For instance, DFA-alpha1 < 0.75 is considered an indicator of exhaustive efforts during exercise [48]. During Rest-1, all three classes showed DFA-alpha1 > 1.0; Class 3 had DFA-alpha1 < 0.7 in Cycling-2 and Classes 2 and 3 both had DFA-alpha1 < 0.7 in Cycling-3. The VLF% values were much higher during Cycling-3 than during other experimental stages (regardless of classes); however, the VLF% values of subjects in Class 3 were higher than those of other subjects in all experimental stages. The sympathetic (LF%) increases and vagal (HF%) decreases tend to be more pronounced for upper limb exercise than for lower limb exercise [49]. Nonetheless, in a situation involving a sharp increase in VLF% values, both HF% and LF% values decreased [50]. The LF% values were higher for Class 1 than for other classes; however, the effect due to an elevated VLF% was not very strong on Class 1. As shown in Figure 1, HF% values of all three classes dropped between Cycling-1 and Cycling-3. As mentioned previously, ApEn values are strongly affected by breathing, that is, the values decrease under slow breathing and increase under rapid breathing [36]. Subjects in Class 3 presented ECG ApEn values that were far higher than that of other subjects across all experimental stages. The Rest-1 ApEn values in Class 2 were lower than that of other subjects and HF% values were higher.

**Figure 2:**
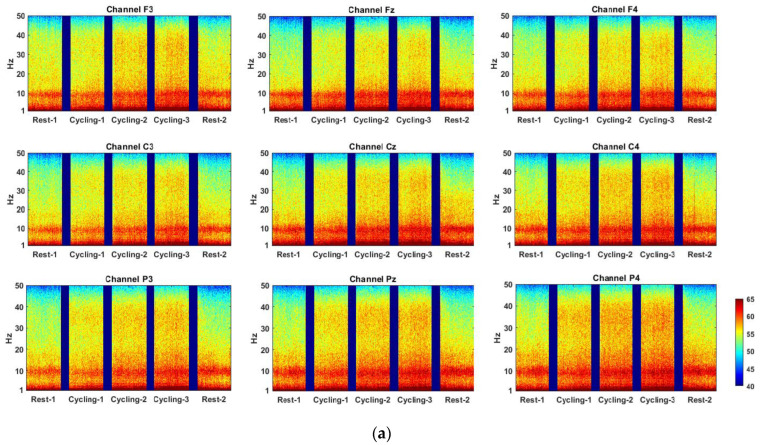

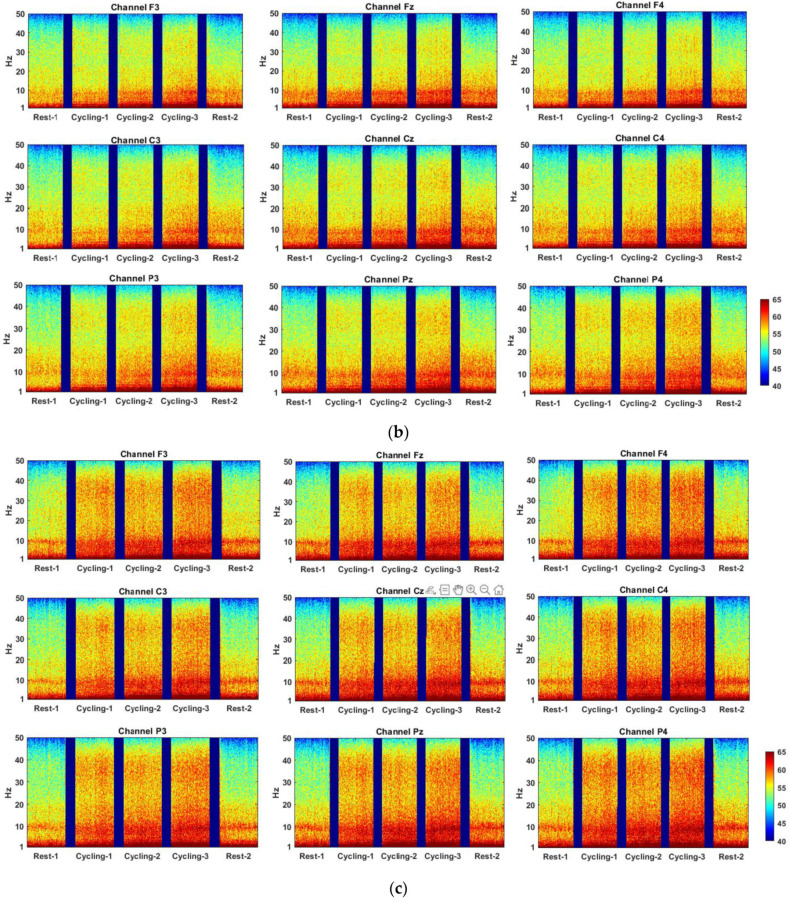
(**a**) EEG power spectral density in the 0.5–50 Hz interval of 9 channels in Class 1 (Note: Y-axis is shown in the 10 log_10_ scale); (**b**) EEG power spectral density in the 0.5–50 Hz interval of 9 channels in Class 2; (**c**) EEG power spectral density in the 0.5–50 Hz interval of 9 channels in Class 3.

**Figure 3:**
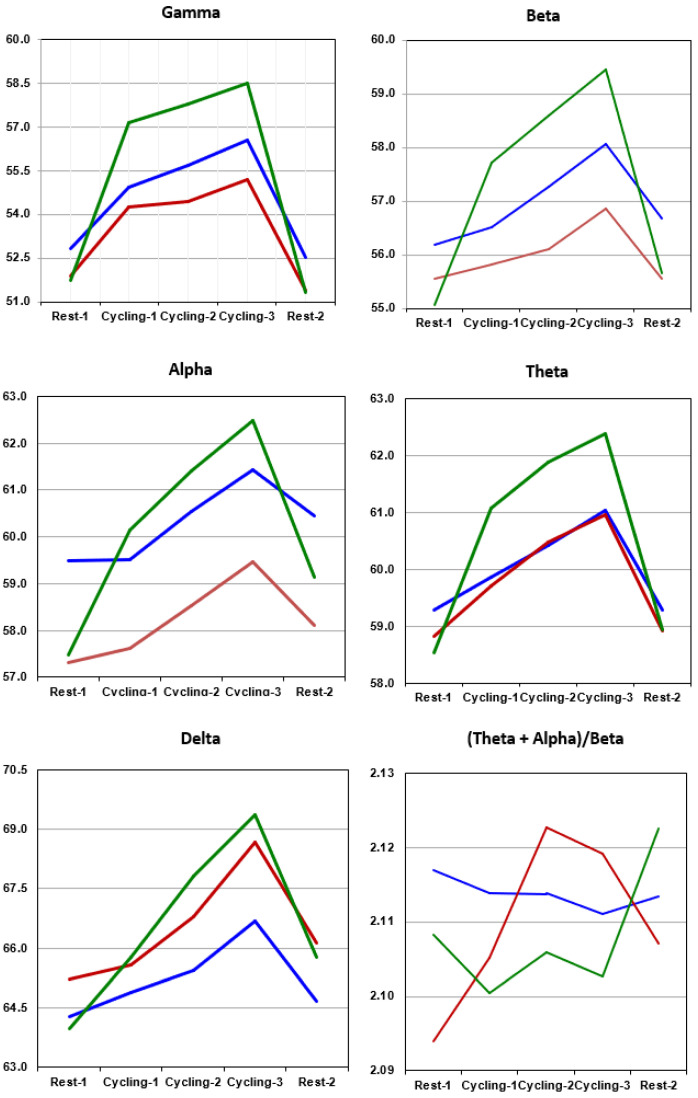
Oscillatory activity in different frequency bands, as observed in the central electrodes (C3, C4 and Cz) under resting-states and during cycling exercise (**blue** = Class 1, **red** = Class 2 and **green **= Class 3). At the bottom of the right panel, the ratio between theta plus alpha and beta powers are depitcted for the 3 classes.

Figure 2 shows the spectral power density from 0.5 to 50 Hz across the time scale within the five experimental stages in the nine channels. The power is presented in the 10 log_10_ scale as computed in EEGLab [51]. The results indicate that the alpha power levels of Classes 1 and 3 were stronger than that of Class 2, particularly during Cycling-3. Figure 3 presents the average power levels in the C3, Cz and C4 channels in the five experimental stages. The average power values in the frontal and parietal channels were similar to those shown in Figure 3. In the gamma, beta and alpha bands, it is interesting to note that the relative EEG power levels in the three classes were roughly the same as the relative ApEn values in Figure 1 in the three cycling stages. In other words, the highest ApEn values as well as alpha, beta and gamma power levels were for Class 3, followed by Class 1 and then by Class 2. These differences in cycling stages among the three classes were statistically significant after controlling for the effects of the same frequency power levels in Rest-1. For instance, Classes 1 and 2 both significantly differed from Class 3 in terms of alpha power in all three cycling stages (*p* < 0.001), after controlling for the effects of the same frequency power levels in Rest-1. Classes 1 and 2 differed significantly from Class 3 in terms of theta powers (*p* < 0.001) and gamma power (*p* = 0.001). Class 1 differed significantly from Class 3 in terms of delta power in Cycling-2 and Cycling-3. Delta activity in Class 2 was similar to that of Class 3 (no significant differences between the two classes in any cycling stages).

GTRs were higher in Class 3 than in other classes. In Rest-1 and Rest-2, the GTR of Class-2 was always far lower than that of other classes, which is consistent with the HF% values in Figure 1. Classes 1 and 3 showed F4 (right) > F3 (left) in alpha power levels in all experimental stages and the degree of asymmetry was larger during Rest-1 and Rest 2 than during the cycling stages. Class 2 always showed F3 > F4 in alpha power levels in all experimental stages. All three classes showed F3 > F4 in beta powers; however, beta-asymmetry was less pronounced in Class 2. Studies have already shown that EEG slow wave bands, particularly frontal theta and delta powers, are associated with cardiac vagal control [52,53]. In the current study, HF% values in Rest-1 were significantly and negatively correlated with degrees of theta asymmetry in all experimental stages. In other words, HF% values in Rest-1 were inversely proportional to theta activity in F4 during cycling stages and Rest-2. In all experimental stages, Classes 1 and 3 showed F4 > F3 in theta power, whereas Class 2 showed F3 > F4. The results pertaining to delta asymmetry were similar to those of theta asymmetry; however, the differences between classes were less pronounced than those with theta activity (i.e., HF% values in Rest-1 were significantly and negatively correlated with delta activity in F4 in Cycling-3 and Rest-2). Figure 3 shows the ratio of (theta + alpha)/beta, which was steadier than the EEG power levels of individual frequency bands. In other words, theta power plus alpha power was double beta power. This ratio was negatively correlated with HF%, particularly for Class 2; however, none of these correlations reached levels of statistical significance.

## 4. Discussion

In experiments, the SDNN and ApEn indices provided similar findings, wherein the heart rate of females in Class 3 was faster than that of other classes and their breathing was more rapid. Females in this group also presented the lowest EEG power levels in Rest-1 and highest EEG power levels during the cycling stages in almost all frequency bands. Higher VLF% values (particularly during Cycling-3) indicate that those females would be prone to periodic breathing if they reached the level of exhaustion during the cycling exercise (e.g., cycling load > 3) [12,13]. Body mass index (BMI) is a simple approach to predicting mortality among the elderly; that is, normal and overweight individuals face a lower risk of mortality than do underweight and obese individuals. Females in Class 3 were nearly underweight, such that they could face an elevated risk of mortality [54]. The proportion of males was higher in Class 1 than in the other two classes. Subjects in Class 1 provided less efforts and presented the lowest average HF% values during the cycling stages. Oscillatory activity in the gamma, beta and alpha bands was higher in Class 1 than in Class 2; however, oscillations in the theta band were similar. Delta power was higher in Class 2 than in Class 1. As mentioned previously, subjects in Class 2 presented more parasympathetic control than did the other subjects (i.e., higher HF% values) in all experimental stages. GTRs in Class 2 were far lower than those in the other two classes. F3 (left) power levels in Class 2 were stronger than F4 (right) in the delta, theta and alpha bands. It is likely that subjects in Class 2 were not suffering as much stress and/or anxiety during the experiment. It is also interesting to note that the HF% values in Rest-1 are a predictor of the relative theta power in F3 and F4 during cycling stages and Rest-2. We hypothesize that stronger F3 theta activity was an indication of more parasympathetic control during the experiment. Nonetheless, it is likely that females in Class 3 were under more stress and/or anxiety during the experiment than the other subjects were.

EEG power levels in mu rhythms (i.e., mu-alpha and mu-beta) are believed to be involved in motor control under the influence of the basal ganglia and sensorimotor functioning [55]. Mu rhythms are sensitive to movement-related and cognitive tasks. Simultaneous recording of EEG and blood oxygen level-dependent (BOLD) signals has revealed that mu power levels are negatively correlated with BOLD in the sensorimotor, attention control and putative mirror neuron networks. Moreover, mu power levels are positively correlated with BOLD in the salience network, including the anterior cingulate cortex and anterior insula [56]. This makes it difficult to link specific functions to mu rhythms. Researchers have previously reported that increased delta activity is predictive of bursts in the heart rate and an EEG indicator of brain–heart coupling [52]. DFA-alpha1 is a strong predictor of delta activity in the subsequent stage. For example, delta activity during Cycling-3 was significantly and negatively correlated with DFA-alpha1 during Rest-1 (*p* = 0.029), Cycling-1 (*p* = 0.001) and Cycling-2 (*p* = 0.035). The signs of exhaustive efforts in Cycling-2 and Cycling-3 were more pronounced in Classes 2 and 3 than in Class 1, as indicated by the relatively low DFA-alpha1 values. This observation could be valuable in interpreting delta activity (see Figure 3).

To summarize, rapid breathing affected the gamma, beta and alpha power levels during cycling exercise. The theta power level was highly associated with stress during cycling exercise and the delta power level revealed the exhaustive effort. ECG ApEn during Rest-1 and the subject’s weight were sufficient to identify nine females for whom EEG power was higher than other subjects across cycling stages. The breathing of those females was more rapid than that of other subjects and their heart rates were faster. The degrees of alpha and theta asymmetry were also higher in those females due, presumably, to elevated stress levels during cycling exercise. It appears that those females faced an elevated risk of periodic breathing and mortality during exhaustive exercise. In addition to HRV parameters during Rest-1, it also appears that EEG asymmetry during cycling exercise may provide valuable information pertaining to the emotional responsiveness of subjects. Delta activity or DFA-alpha1 can be used to predict bursts in the heart rate and exhaustive efforts. Both delta activity and DFA-alpha1 could be used in conjunction with ECG VLF% values to monitor elderly adults facing an elevated risk of heart failure during exhaustive exercise.

One limitation of this study was the small sample size, such that the range of BMI scores was limited. This may explain why body weight was significantly interacted with Rest-1 ApEn, rather than BMI. Another limitation was a lack of patients dealing with chronic heart failure, by which to establish a baseline for the classification of healthy and at-risk adults based on HRV and EEG power levels. Thus, our interpretation of Rest-1 ApEn, DFA-alpha1 and VLF% values for Class 3 should be considered tentative, requiring further validation in larger samples. In conclusion, the resting ECG ApEn index is recommended for screening the high-risk elderly prior to exercise interventions. Other resting-state HRV indices remain to be validated for their use in predicting the risk of heart failure, especially in clinical patients. 

## Data Availability

Empirical data are available upon request from Professor Yu-Te Wu and the corresponding author.
